# Ultrathin Si/CNTs Paper-Like Composite for Flexible Li-Ion Battery Anode With High Volumetric Capacity

**DOI:** 10.3389/fchem.2018.00624

**Published:** 2018-12-13

**Authors:** Jinzhou Fu, Hao Liu, Libing Liao, Peng Fan, Zhen Wang, Yuanyuan Wu, Ziwei Zhang, Yun Hai, Guocheng Lv, Lefu Mei, Huiying Hao, Jie Xing, Jingjing Dong

**Affiliations:** ^1^Beijing Key Laboratory of Materials Utilization of Nonmetallic Minerals and Solid Wastes, National Laboratory of Mineral Materials, School of Materials Science and Technology, China University of Geosciences Beijing, China; ^2^School of Science, China University of Geosciences Beijing, China

**Keywords:** Si electrodeposition, carbon nanotubes, volumetric capacity, anode, flexible battery

## Abstract

Thin and lightweight flexible lithium-ion batteries (LIBs) with high volumetric capacities are crucial for the development of flexible electronic devices. In the present work, we reported a paper-like ultrathin and flexible Si/carbon nanotube (CNT) composite anode for LIBs, which was realized by conformal electrodeposition of a thin layer of silicon on CNTs at ambient temperature. This method was quite simple and easy to scale up with low cost as compared to other deposition techniques, such as sputtering or CVD. The flexible Si/CNT composite exhibited high volumetric capacities in terms of the total volume of active material and current collector, surpassing the most previously reported Si-based flexible electrodes at various rates. In addition, the poor initial coulombic efficiency of the Si/CNT composites can be effectively improved by prelithiation treatment and a commercial red LED can be easily lighted by a full pouch cell using a Si/CNT composite as a flexible anode under flat or bent states. Therefore, the ultrathin and flexible Si/CNT composite is highly attractive as an anode material for flexible LIBs.

## Introduction

Flexible electronic products, such as wearable electronics, smart textiles, rollup displays, bendable mobile phones, and implantable medical devices, are developing at a tremendous pace (Stoppa and Chiolerio, 2014; Zhou et al., 2014; Pu et al., 2015; Liang et al., 2017a,b, 2018). Therefore, thin and lightweight flexible lithium-ion batteries with high gravimetric and volumetric capacities are increasingly becoming required to meet the characteristics of these flexible electronic products that can be bendable and foldable (Wang et al., 2014; Liu et al., 2017b). However, the traditional method for LIBs' electrode preparation, being tape-casted on a flat current collector method, is not suitable for the preparation of flexible electronic devices. Several new methods and technologies, such as chemical vapor deposition (Magasinski et al., 2010; Hu et al., 2011; Wang et al., 2017), electrospinning (Xu, 2015; Kim et al., 2016b), and electrodeposition (Hu et al., 2010; Gwon et al., 2011; Xiao et al., 2014b; Ma et al., 2016), were developed to achieve flexible battery electrodes. Among these new methods, the electrodeposition of electroactive materials on flexible current collectors is promising for flexible energy storage systems due to its simple operation and low cost. For example, Chou et al. reported that MnO_2_ nanowires can be electrodeposited on carbon nanotube as free-standing, flexible electrode for supercapacitors (Chou et al., 2008). Li et al. electrodeposited nickel sulfide on graphene-covered cotton as a flexible supercapacitor electrode (Li et al., 2015).

As a typical anode material, Si has attracted much attention for the flexible LIBs due to its high specific capacity, which is nearly 10-fold higher than that of graphite (Jung, 2003; Chan et al., 2008; Szczech and Jin, 2011; Choi and Aurbach, 2016). However, the larger storage ability of Li atoms brings a severe volumetric change (>300%), leading to the pulverization of electrodes and a subsequent loss of electrical contact between the Si-active material and the current collector (Ryu et al., 2004; Szczech and Jin, 2011). Construction of nanostructured Si is an effective way to overcome the severe volume changes of Si anodes because abundant interspace and/or voids in the nanostructured configurations can accommodate the volume expansion of Si during lithiation. Therefore, the configurations of nanostructured Si directly grown on flexible current collectors are usually employed for Si-based flexible batteries (Li et al., 2014; Ma et al., 2016; Liu et al., 2017b; Wang et al., 2017; He et al., 2018). In addition, these free-standing composites are free of binders/additives, which are usually inactive and can reduce the capacity of the whole electrode. Moreover, the strain introduced during lithiation/delithiation can be relaxed further by the ductile current collectors (Yu et al., 2012). In those configurations, carbon-based materials, such as carbon nanotubes (CNTs), carbon fibers and graphene, are selected as flexible current collectors due to their high electronic conductivity and excellent mechanical properties (Falvo et al., 1997; Chew et al., 2009; Landi et al., 2009; de las Casas and Li, 2012; Volder et al., 2013). For example, Fu et al. synthesized an aligned CNT-Si sheet structure as a binder-free and flexible anode (Fu et al., 2013). Weng et al. reported a novel three-dimensional aligned CNT/Si architecture for flexible anodes (Weng et al., 2014). Xiao et al. reported stable Si/CNT coaxial nanofiber composite on a copper current collector through a simple layer-by-layer assembly method (Xiao et al., 2014a). The above carbon-based current collectors serve as not only the conductive network but also a flexible buffer matrix to accommodate the volume change of Si during the lithiation/delithiation processes, thus improving the electrochemical performance of Si-based anodes. Nevertheless, the above synthetic methods usually involved expansive equipment and/or toxic and flammable gases. Moreover, a significant non-unformal Si deposited layer was often formed when substrates had a complex three-dimensional structure due to the shadow effect (Karunasiri et al., 1989). Therefore, it is necessary to find a simple and low-cost deposition method of Si. Electrodeposition is promising, in which amorphous or polycrystalline Si can be produced by electrochemical reduction of Si precursors in an organic solvent (Munisamy and Bard, 2010; Nara et al., 2012; Osaka et al., 2014) or ionic liquid (Zhou et al., 2011) at ambient temperature. What is more, the Si deposit can be conformally coated on a three-dimensionally conductive substrate (Liu et al., 2013). Nevertheless, to the best of our knowledge, the electrochemical performance of the electrodeposited Si has not been explored as a flexible anode material for LIBs.

In the present work, we report a paper-like free-standing flexible composited anode for LIBs with high volumetric capacity. This composite was realized by the conformal electrodeposition of a thin layer of Si on carbon nanotube film. In this configuration, CNT film was selected as a flexible current collector due to its excellent mechanical properties, high electronic conductivity, high porosity, and light weight (Falvo et al., 1997; Chew et al., 2009; Landi et al., 2009; de las Casas and Li, 2012; Volder et al., 2013). The large volume expansion of Si during the lithiation process can be accommodated by the porous structure of CNT film. The flexible Si/CNT composite exhibited very high volumetric capacities in terms of the total volume of the whole electrode (including the active material and the current collector), surpassing the most previously reported Si-based flexible electrodes. In addition, a pouch cell using Si/CNT composite as a flexible anode was assembled and a commercial red LED can be easily lighted by this pouch cell under flat or different bent states. Therefore, the ultrathin and flexible Si/CNT composites synthesized by electrodeposition are highly attractive as anodes for flexible LIBs.

## Experimental Section

### Preparation of the Si/CNTs Paper-Like Composite

First, the thin CNT film (CNTs, 10 × 10 mm, ~20 μm thick, Suzhou TANFENG Tech Co., Ltd.) was ultrasonically cleaned with deionized water and alcohol for 15 min, then dried at 70°C in a vacuum oven for 12 h. A thin layer of gold was deposited on the CNT film using thermal evaporation (ZHD-400, Beijing Technology Co., Ltd.) to further improve the conductivity of the CNTs. Next, Si was electrodeposited on the CNT substrate in a non-aqueous electrolyte solution using a three-electrode system (Nicholsonz, 2005; Nishimura and Fukunaka, 2007; Munisamy and Bard, 2010; Liu et al., 2013; Osaka et al., 2014). The CNT film and Pt foil were employed as the working electrode and counter electrode, while a Pt wire was used as the quasi-reference electrode. A solution of 0.5 M silicon tetrachloride (SiCl_4_, 99.998%, Sigma-Aldrich) and 0.1 M tetrabutylammonium chloride (TBACl, >98.0%, TCI) in 20 ml propylene carbonate (PC, anhydrous, 99.7%, Sigma-Aldrich) was used as the electrolyte. The electrodeposition of Si was operated under a current density of −3 mA cm^−2^ at ambient temperature in an argon-filled glove box (O_2_ < 0.1 ppm, H_2_O < 0.1 ppm,). Then, the obtained samples were dried and annealed at 350°C for 30 min in the glove box. The mass of Si deposits was obtained using electronic analytical balance (BSA 124S, Sartorius).

### Characterizations

The stress-strain curves of the samples were tested by the tensile test (Instron 5848 MicroTester). The crystallinity was examined by X-ray diffraction (XRD, D8 Advance, Bruker) with Cu Kα radiation (λ = 1.5406 Å). The morphologies and the chemical compositions were characterized by a scanning electron microscope (SEM, ZEISS, Merlin) and related energy-dispersive X-ray detector (EDX, ZEISS, Merlin). A transmission electron microscope (TEM, Tecnai G2 F20 S-TWIN TMP, FEI) equipped with an energy-dispersive X-ray detector was also used.

### Electrochemical Performance of the Si/CNTs Electrode

Two thousand and thirty two coin-type half cells were used to examine the electrochemical performance of the samples. During the cell assembly, binder, and conducting carbon were not used. 1.0 M LiPF6 in ethylene carbonate/dimethyl carbonate/fluoroethylene carbonate solvent was used as the electrolyte. Galvanostatic charging/discharging cycles were tested between 0.01 and 2.0 V under various rates on a multichannel battery test system (CT-4008, NEWARE Technology Ltd.). The electrochemical impedance spectroscopies (EIS) were tested using an electrochemical workstation (Vertex, Ivium, Brillante) in the frequency range from 100 kHz to 1 Hz. The morphologies of the electrodes after cycling testing were monitored and a dilute acetic acid solution was used to remove the surface-electrolyte interphase (SEI) layer on the electrode (Choi et al., 2010). For prelithiation, the Si/CNT electrode contacted with Li foil for 30 min. A small amount of battery electrolyte was dropped into the gap between the Si/CNT electrode and Li foil. For the demonstration of flexible full battery, the Si/CNT composite electrode as a flexible anode and conventional LiFePO4 cathode (a mixture of 80% LiFePO4 powder, 10% carbon black and 10% poly(vinylidene fluoride) pasted on Al foil) were packaged using Al plastic film to make a pouch cell.

## Results and Discussions

### Electrodeposited Si on CNTs and Related Characterizations

**Figure 1:**
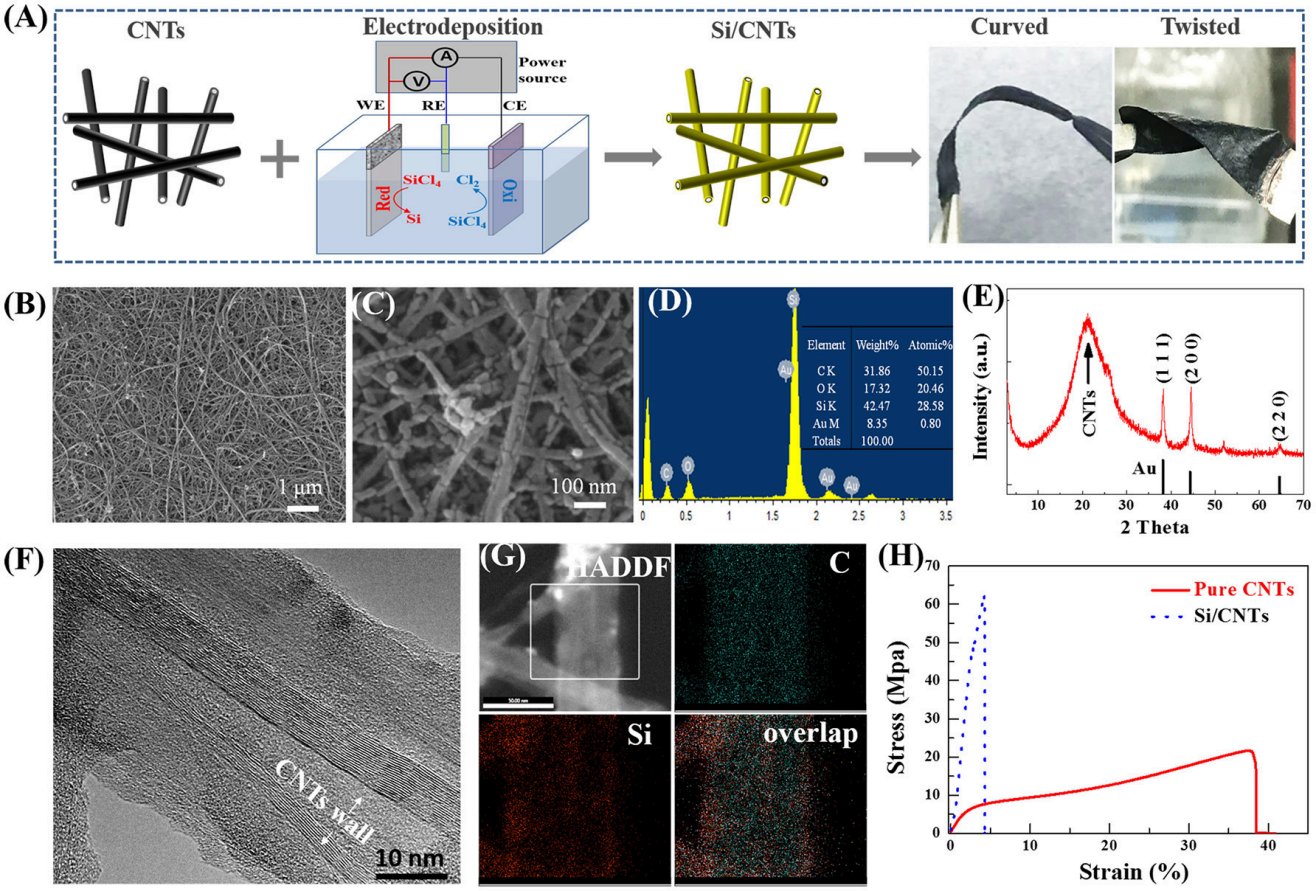
**(A)** Schematic of the synthesis of the Si/CNTs and the photographs of this composite at curved and twisted states. **(B,C)** Plan-view SEM images of Si/CNT composite at different magnifications. **(D)** EDX spectrum taken from this composite. **(E)** XRD spectrum of the Si/CNT composite. **(F)** High resolution TEM images taken from a typical part of the Si/CNT composite, and **(G)** EDX elemental maps taken from the sample region shown in high angle annular dark filed (HAADF) image of the part of the nanotube. **(H)** Stress-strain curve of typical CNTs and the above composite.

Si was electrodeposited on the CNT substrate in a non-aqueous electrolyte solution using a three-electrode system at ambient temperature as shown in Figure 1A. This method is quite simple and easy to scale up and has a low cost compared to other deposition techniques, such as sputtering or CVD. Linear sweep voltammetry of the electrodeposition system was first carried out with and without SiCl_4_ in the electrolyte, with the results shown in Figure S1A (Supporting information). There are no obvious reduction peaks in the linear sweep voltammogram of the system without SiCl_4_. In contrast, a wide hump located at around 1.5 V in the linear sweep voltammogram is observed after SiCl_4_ added in the electrolyte, indicating the electro-reduction of SiCl_4_ to Si. During the preparation of the Si/CNTs composite, a constant current density of −3 mA cm^−2^ was employed for Si electrodeposition. The corresponding electrodeposited potential maintains around 2.4 V (Figure S1B, Supporting information). The mass loading of the Si deposit can be controlled by changing the electrodeposition time (Figure S1C). For example, the mass loading of silicon was about 1.1 and 1.5 mg cm^−2^ for 4 h and 6 h electrodeposition (denoted as Si/CNTs-4 and Si/CNTs-6), respectively. The above Si/CNT composites show good flexibility. For example, they can be curved or twisted many times without any damage (Figure 1A).

In order to reveal the detailed microstructure of the composite, scanning electron microscopy (SEM) of Si/CNT-4 was carried out, with results shown in Figures 1B,C. One can observe that carbon nanotubes intertwine to form a porous network. After electrodeposition, a thin layer of Si with a thickness of ~10–20 nm is conformally deposited on the wall of CNTs (Figure 1C) rather than the external surface of the composite. In addition, the morphology of Si/CNT-6 was similar with that of Si/CNTs-4 (Figure S1, Supporting Information). Because the Si deposit mainly occupied the void of the CNTs paper, the thickness of the composites was maintained at ~20 μm after Si deposition (Figure S1D). The EDX indicates that Si/CNT is composed of C, Au, Si, and O elements (Figure 1D). The O element possibly originates from the decomposition of the organic electrolyte during the electrodeposition (Nara et al., 2012) as well as the oxidation of the sample which was exposed to air during the sample transfer process (Nicholsonz, 2005; Nishimura and Fukunaka, 2007; Nara et al., 2012).

The crystallinity of the deposit during the electrodeposition was investigated by XRD, with results shown in Figure 1E. A typical wide hump at ~21° is observed, resulting from the amorphous CNT substrate (Chew et al., 2009). In addition, three diffraction peaks located at 38.2, 44.4, and 64.6° correspond to the (111), (200), and (220) planes of Au, respectively (Au was deposited on CNTs to increase the conductivity). No other diffraction peaks are observed, revealing that the Si deposit is amorphous.

The structural information of individual nanowires was further carried out by TEM. Here, the Si/C composite without gold layer was used for the preparation of the TEM sample because it was difficult to obtain a good TEM sample from Si/C composite with gold layer. This is probably due to the multilayer structure of the Si/C composite with gold being more easily destroyed during the preparation of the TEM sample (scratch some fibers from the composite). Figure 1F shows the most representative image of the sample. The surface of the composite is rougher after Si electrodeposition. The CNT walls are clearly observed and the shell region with thickness of ~10 nm appears amorphous in the higher magnified TEM image (Figure 1G), being consistent with the above XRD results. The spatial distributions of the C and Si elements are revealed by the elemental mapping (Figure 1G). Because partial organic electrolytes can be decomposed during the electrodeposition process (Nara et al., 2012), the C and Si elements are distributed over the whole sample.

Tensile tests were carried out to understand the mechanical properties of the composite. Figure 1H shows the corresponding stress-strain curves of the CNTs and composites. The CNT paper exhibits three distinct deformation stages, i.e., linear-elastic region, plastic region, and fracture region. In the linear-elastic region, the strain ε is <1.5%, with an elastic modulus of ~335 MPa. When the stress increased from 4.5 to ~20 MPa, the strain was in the range of 1.2 to 37.7%, corresponding to the plastic behavior. A fracture happens when ε is higher than ~38%. In contrast, the strain of the composite increases almost linearly to ~2.7% with the stress increased to ~48 MPa. The elastic modulus is ~1,778 MPa, much higher than that of pristine CNTs. Nevertheless, the plastic regions with 2.7% < ε < 4.2% are small, indicating the composite is more brittle than pristine CNT paper. The high deformation ability of the CNT paper originates from CNTs itself, which usually has high strain before fracture (Falvo et al., 1997). After electrodeposition, the brittle silicon deposit strongly adheres to the walls of CNTs, leading to much higher tensile strength of the composite as compared to pristine CNT paper. Nevertheless, the composite still exhibits good flexibility, as shown in Figure 1A.

### Electrochemical Performance of Si/CNTs Electrodes

**Figure 2:**
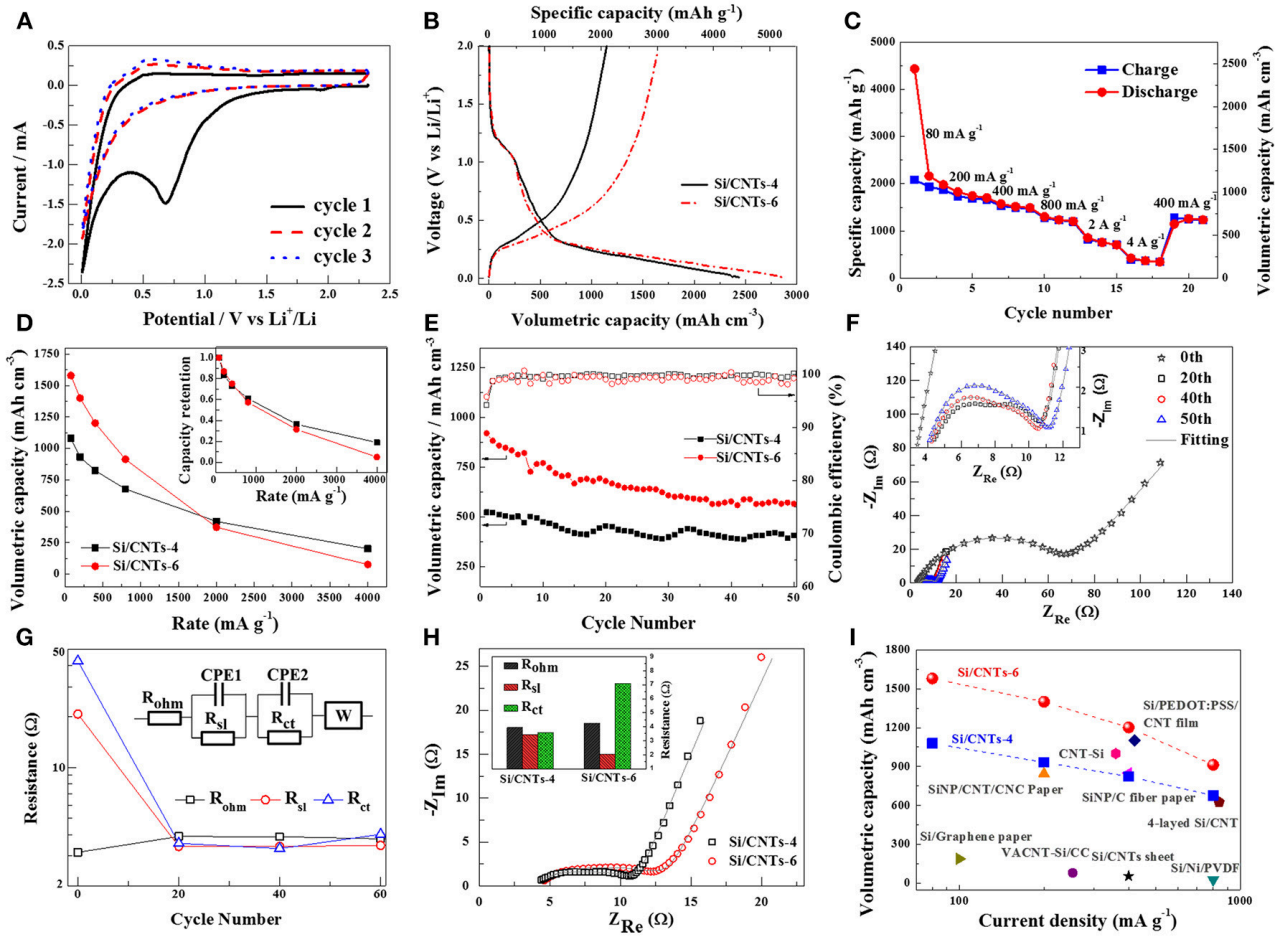
**(A)** Cyclic voltammogram of Si/CNT-4. **(B)** Voltage profile of Si/CNT-4 and Si/CNT-6 electrodes during the first cycle under 80 mA g^−1^. **(C)** The discharge/charge capacities of this electrode at different rates. **(D)** The charge volumetric capacities of the Si/CNT-4 and Si/CNT-6 at different rates. The comparison of the volumetric capacity retention of these three Si/CNT electrodes are shown in the inset. **(E)** Cycling performance of these two electrodes at the current density of 800 mA g^−1^. **(F)** Nyquist plots of the Si/CNT electrodes at initial and ~0.5 V vs. Li/Li^+^ during 20th−60th charging process. The inset shows the enlarged plots in high frequency range. **(G)** Comparison of the individual real impedance, R_ohm_, R_sl_, and R_ct_ obtained from the Nyquist plots. The inset shows the equivalent circuit model for this electrode. R_ohm_ stands for series ohmic resistance. R_sl_ and CPE1 represent surface layer resistance and capacitance, respectively. R_ct_ and CPE2 represent the charge transfer resistance and double layer capacitance. W represents Warburg impedance. The lines in **(F)** are the fitted curves using this model. **(H)** Nyquist plots of these Si/CNTs electrodes at ~0.5 V during the 20th charging process. The inset shows the comparison of the individual real impedance, R_ohm_, R_sl_, and R_ct_ obtained from the Nyquist plots. **(I)** Comparison of the volumetric capacities of the Si/CNT composite with Si-based electrodes reported as flexible in the literature. The details of these references are summarized in Table S1.

The electrochemical properties of the Si/CNT composite were examined using half-cell. Figure 2A shows the cyclic voltammogram of the Si/CNT-4. During the cathodic scan, a wide hump of around 0.7 V vs. Li/Li^+^ was observed in the first discharging process, corresponding to the formation of the SEI layer. This is consistent with previous lithiation profiles. This hump disappears in the next discharging process, suggesting that the SEI layers were mainly formed in the first lithiation process. In the following charging process, two oxidation peaks can be observed at ~0.28 and ~0.51 V vs. Li/Li^+^, corresponding to the Si delithiation. Figure 2B shows the voltage profile of Si/CNT-4 during the first discharging/charging at the current density of 80 mA g^−1^. A sloping curve below 0.35 V vs. Li/Li^+^ was observed during the lithiation process, which is a typical lithiation behavior of amorphous Si (Jung, 2003). In addition, a wide hump is observed around 1.25 V vs. Li/Li^+^ in the first lithiation profile, resulting from the electrolyte decomposition to form the SEI layer (Ruffo et al., 2009; Kim et al., 2016a). Volumetric capacity is important for the practical LIBs. We calculated the volumetric capacity of the flexible Si/CNT composite in terms of whole volume of the free-standing composited electrode (including Si deposit and CNTs current collector). The discharging and charging volumetric capacities of Si/CNT-4 in the first cycle are 2,438 mAh cm^−3^ (4,434 mAh g^−1^) and 1,146 mAh cm^−3^ (2,084 mAh g^−1^), respectively, corresponding to an initial coulombic efficiency of ~47%. The larger initial irreversible capacity results from the surface reaction to form the SEI layer (Szczech and Jin, 2011; Liu et al., 2013). Nevertheless, the coulombic efficiencies increase to ~99% in the next few cycles. Figure 2C plots the charging capacities (delithiation) of Si/CNT-4 composite at different rates. At a low current density of 80 mA g^−1^, the charging volumetric capacity is ~1,079 mAh cm^−3^ (1,962 mAh g^−1^). While the charging current densities increase to 200 mA g^−1^, 400 mA g^−1^, 800 mA g^−1^, 2 A g^−1^, 4 A g^−1^, the charging volumetric capacities decrease to 932 mAh cm^−3^ (1,694 mAh g^−1^), 823 mAh cm^−3^ (1,497 mAh g^−1^), 677 mAh cm^−3^ (1,231 mAh g^−1^), 420 mAh cm^−3^ (763 mAh g^−1^), and 202 mAh cm^−3^ (368 mAh g^−1^). Nevertheless, the charging volumetric capacity can be recovered to ~688 mAh cm^−3^ (1,251 mAh g^−1^) when the charging current density returns to 400 mA g^−1^. We further examined the electrochemical properties of the pristine CNT paper, with the results shown in Figure S2. The pristine CNT paper exhibited a much lower capacity (<150 mA h g^−1^) than the composite, indicating that the capacities of the composites were mainly contributed by the Si deposit.

In order to increase the volumetric capacities of the Si/CNT composites, more Si deposit was grown on the CNT substrate by extending the deposited time from 4 to 6 h. The shape of the charging/discharging curves of the Si/CNTs-6 are similar to those of Si/CNT-4 (Figure 2B), but the discharging/charging volumetric capacities at the current density of 80 mA g^−1^ increase to 2,856 mAh cm^−3^ (3,808 mAh g^−1^) and 1,655 mAh cm^−3^ (2,206 mAh g^−1^), respectively. As a result, the initial coulombic efficiency of the Si/CNTs-6 is improved to ~58%, higher than that of the Si/CNTs-4 electrode (~47%). Nevertheless, the increments of the charging volumetric capacities progressively decrease with the increase of the charging rates in the range of 80~1,740 mA g^−1^ (Figure 2D). At higher rates, the volumetric capacity of Si/CNT-6 becomes lower than that of Si/CNT-4. We further plotted the capacity retentions of these composite electrodes (inset of Figure 2D) for better comparison, revealing that the rate performance of the Si/CNT composites declines with the increase of the mass of Si deposit.

Figure 2E shows the cycling performance of these two electrodes. The initial volumetric capacities of Si/CNT-4 and Si/CNT-6 at a charging/discharging current density of 800 mA g^−1^ are 539 mAh cm^−3^ and 920 mAh cm^−3^, while decay to 408 mAh cm^−3^ and 567 mAh cm^−3^, respectively, after 50 cycles. This reveals that Si/CNT-4 is more stable than Si/CNT-6. Nevertheless, the coulombic efficiencies of both electrodes remain at ~99% during the cycling test. The morphology of the composite after the cycles (SEI layer was partially removed with dilute glacial acetic acid) was further characterized using SEM, with results shown in Figure S3. This composite showed a porous structure and Si was still observed in the EDX spectrum, indicating that the main structure of the Si/CNT composite was maintained. On the other hand, it should be noted that surface engineering or coating was an effective way to improve the cycling stability of the Si-based electrode (Yi et al., 2013; Liu et al., 2014; Li et al., 2016). Therefore, the cycling stability of the Si/CNT composite could be enhanced by coating a thin layer of carbon or non-active materials using pulsed laser deposition or CVD. Nevertheless, this part is out of the main scope of the present work and will be done in future work.

In order to understand the changes of the Si/CNT electrode during the cycling test, the electrochemical impedance spectrum (EIS) measurements were operated as initially and at ~0.5 V vs. Li/Li^+^ during the 20th to 50th charging process. The corresponding Nyquist plots of Si/CNT-4 are shown in Figure 2F. Two partially mixed depressed semicircles related to the resistance of the SEI layer and charge transfer resistance between the electrode/electrolyte interfaces can be observed in the high and medium frequency range, respectively. In the low frequency range, an inclined line corresponding to the Li ion diffusion in the solid electrode can be found (Liu et al., 2017a). The electrical parameters R_ohm_, R_sl_, and R_ct_ can be obtained by simulation of the Nyquist curves using an equivalent circuit model (Figure 2G). During the cycling, the ohmic resistances of the Si/CNT composite are quite stable. Nevertheless, the SEI layer resistance and charge transfer resistance drop sharply after discharging/charging processes, resulting from the activation of pristine Si electrodes. After that, these two resistances slowly increase during the 20th to 50th cycles, indicating the SEI layer became thick during the cycling. Therefore, the volumetric capacities gradually decrease during the cycling (Figure 2E) due to continual loss of fresh Si at the electrode's surface in the formation of the SEI layer.

In order to reveal the difference between the Si/CNT-4 and Si/CNT-6 electrodes, EIS measurements were performed at ~0.5 V during the 20th charging process (Figure 2H). It can be found that the ohmic resistances are almost same, but the SEI layer resistances decrease and the charge transfer resistances increase from Si/CNT-4 to Si/CNT-6. As the deposition time increased, the thickness of Si deposit on the walls of CNTs became thicker, reducing the porosity and the surface area of the Si/CNT composites. Thus, the formation of the SEI layer was inhibited partially, leading to the resistance of the surface layer being decreased and the first coulombic efficiency then improved in the Si/CNT-6 (Figure 2A). On the other hand, the charge transfer resistance increases with the increase of the thickness of the Si deposit, resulting in a worse rate performance from the Si/CNT-6 as compared to that of the Si/CNT-4 (Figure 2D).

To better evaluate the electrochemical performance of the flexible Si/CNT composite, we further compared the volumetric capacities of the Si/CNT-4 and Si/CNT-6 with Si-based electrodes that were reported in the literature to be flexible (in terms of the total volume of active materials and current collector; Cui et al., 2010; Fu et al., 2013; Chen et al., 2014; Xiao et al., 2014a,b; Wang et al., 2015, 2016; Xu, 2015; Biserni et al., 2016; Jiang et al., 2016), with results shown in Figure 2I and Table S1. Among the compared Si-based electrodes, the Si/CNT-6 shows the highest volumetric capacity at the various current densities. Furthermore, the Si/CNT composite was prepared using electrodeposition with simple operation and at a low cost, in comparison to the other complex and costly methods involving chemical vapor deposition (Fu et al., 2013; Xiao et al., 2014a; Wang et al., 2016) or etching (Jiang et al., 2016). Therefore, our Si/CNT composites are highly competitive for flexible LIBs as ultrathin and flexible anode materials.

### Prelithiation of Si/CNT Composites and Demonstration of Light-Emitting-Diode Lighting by a Flexible Full Battery

**Figure 3:**
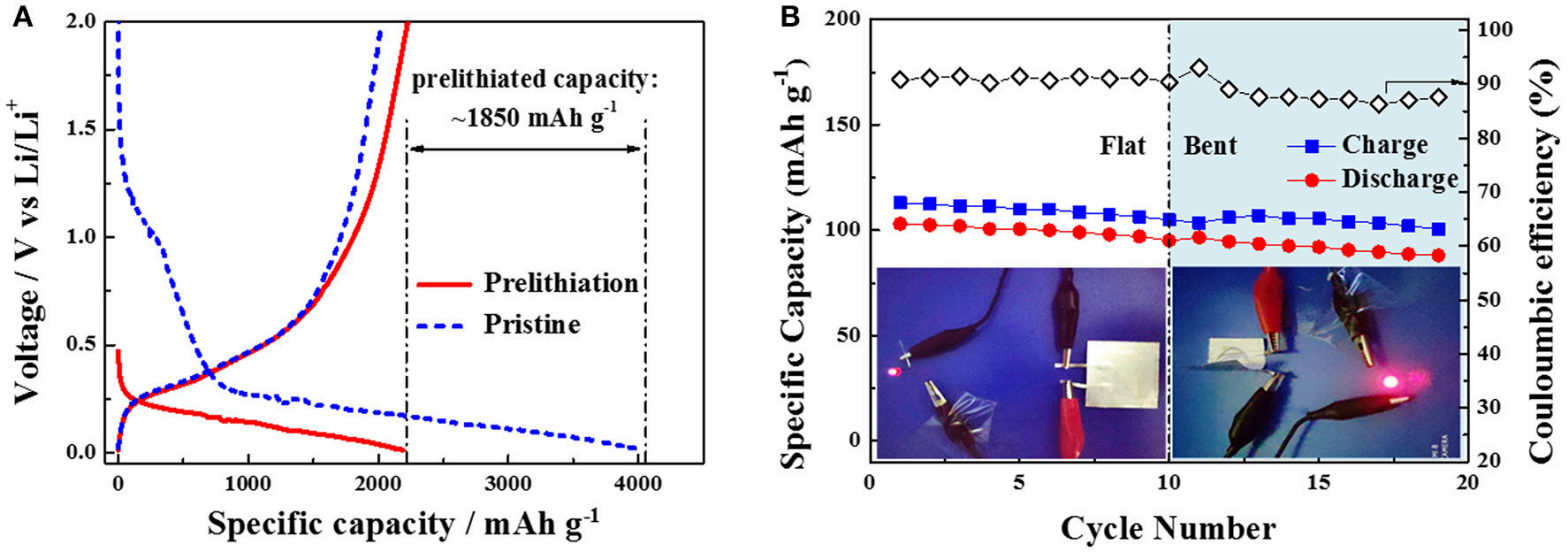
**(A)**Comparison of first discharging/charging profiles of the pristine and prelithiated Si/CNT-4 at the current density of 80 mA g^−1^. **(B)** Cycling performance of a full pouch cell with Si/CNT composite anode and LiFePO_4_ cathode under flat and bent states at current density of 170 mA g^−1^. The inset shows the photos of a red LED lighting by this pouch cell under flat and bent states.

Although the above flexible Si/CNT composites display very high volumetric capacities, their initial coulombic efficiencies are quite low (<60%). This is harmful to practical full LIBs. In order to improve the initial coulombic efficiency, we performed the prelithiation treatment on the Si/CNT-4 as an example by directly contacting this composite with lithium foil for 30 min (a small quantity of lithium battery electrolyte was filled between the composite and Li foil; Liu et al., 2011). Figure 3A shows the first discharging/charging profiles of the pristine and prelithiated Si/CNTs-4 at the current density of 80 mA g^−1^. After prelithiation, the open circuit voltage of the Si/CNT's electrode reduces to ~0.47 V vs. Li/Li^+^ due to the insertion of lithium. In addition, the wide hump around 1.25 V vs. Li/Li^+^ in the first lithiation profile disappears in the prelithiated electrode, indicating that the SEI layer was formed in the prelithiation process rather the first discharging process. Therefore, the first specific discharge capacity of the prelithiated Si/CNT electrode is ~2,188 mAh g^−1^, much less than that of the pristine electrode (~4,038 mAh g^−1^). The first charging capacities of the pristine and prelithiated Si/CNT electrodes are similar. The prelithiation capacity can be calculated as ~1,850 mAh g^−1^. As a result, the initial coulombic efficiency of the prelithiated Si/CNT-4 electrode is improved to 102%, much higher than that of the pristine sample (~47%). Therefore, the poor initial coulombic efficiency problem of the Si/CNT composite can be solved using the prelithiation method. Furthermore, the specific capacities at various charging/discharging rates are also increased (Figure S4, Supporting Information). For example, the specific capacity of Si/CNT-4 at a current density of 800 mA g^−1^ was improved from 1,231 to 1,484 mAh g^−1^ after prelithiation treatment. The improvement of the capacity probably results from the increase of electronic conductivity during the formation of Li_x_Si alloy in the prelithiated Si sample (Pollak et al., 2007).

In order to further demonstrate the flexibility of the Si/CNTs composite for flexible battery, a full pouch cell was assembled with prelithiated Si/CNTs-4 as anode and LiFePO_4_ pasted on Al foil as cathode. The preliminary results show that the specific charging/discharging capacities in terms of the LiFePO_4_ mass of this pouch cell at flat state are ~110 and ~100 mAh g^−1^, respectively, while decreasing a little at a bent state (Figure 3B). In addition, the discharging voltage profile of the pouch cell under the bent condition drops slightly compared to that of the cell under the flat state (Figure S5). The coulombic efficiency is ~91% in the first cycle, which is greatly improved compared to the half-cell of the pristine Si/CNT-4. The following coulombic efficiencies rested at ~90%, which is relatively low for a practically full battery. Nevertheless, this problem may be overcome by optimizing the processes of prelithiation of Si/CNT composites and the assembly of the pouch cell. Furthermore, a commercial red LED can be easily lighted by this pouch cell at flat or different bent states (Figure 3B), demonstrating that Si/CNT composites can be used in flexible batteries.

## Conclusions

In conclusion, ultrathin and flexible Si/CNT composites were fabricated using electrodeposition. Although the composites showed much higher tensile strength as compared to pristine CNT paper, the composites can still be bent or twisted many times without damage, indicating their good flexibility. The flexible Si/CNT composites exhibited high volumetric capacities in terms of the total volume of active material and current collector at various charging/discharging current densities. For example, Si/CNT-6 showed the highest volumetric capacity, ~1,400 mAh cm^−3^, at a current density of 200 mA g^−1^. When the current densities rise, the volumetric capacities of the Si/CNT composite also surpass the most previously reported Si-based flexible electrodes. In order to make a full battery, the poor initial coulombic efficiency of the Si/CNT composites was first solved by prelithiation treatment. We further demonstrated that a commercial red LED can be easily lighted by a pouch cell under flat or bent states. Therefore, the ultrathin and flexible Si/CNT composites are highly promising for their potential application as anode materials in flexible LIBs.

## Author Contributions

HL and LL designed this project. JF carried out the material preparation and electrochemical test. JF, HL, PF, ZW, YW, ZZ, and YH carried out and analyzed the XRD and SEM results. GL, LM, HH, JX, and JD carried out and analyzed the TEM results. JF and HL wrote the paper. All authors discussed the results and revised the manuscript.

### Conflict of Interest Statement

The authors declare that the research was conducted in the absence of any commercial or financial relationships that could be construed as a potential conflict of interest.
