# Supplementary material for: Ultrathin Si/CNTs Paper-Like Composite for Flexible Li-Ion Battery Anode With High Volumetric Capacity
Source: Front Chem. 2018 Dec 13;6:624. doi: 10.3389/fchem.2018.00624 (PMC6300474; doi:10.3389/fchem.2018.00624)
Supplement: Supplementary file 1 [file Data_Sheet_1.docx]

Supplementary Material

Ultrathin Si/CNTs paper-like composite for flexible Li-ion battery anode with high volumetric capacity

Jinzhou Fu ^1^, Hao Liu ^2^*, Libing Liao ^1^*, Peng Fan ^1^, Zhen Wang ^2^, Yuanyuan Wu ^2^, Ziwei Zhang ^1^, Yun Hai ^1^, Guocheng Lv ^1^, Lefu Mei ^1^, Huiying Hao ^2^, Jie Xing ^2^, Jingjing Dong ^2^

*** Correspondence:**Dr. Hao Liu, Email:liuhao1398@cugb.edu.cn;

Dr. Libing Liao, Email:lbliao@cugb.edu.cn

# Supplementary Data


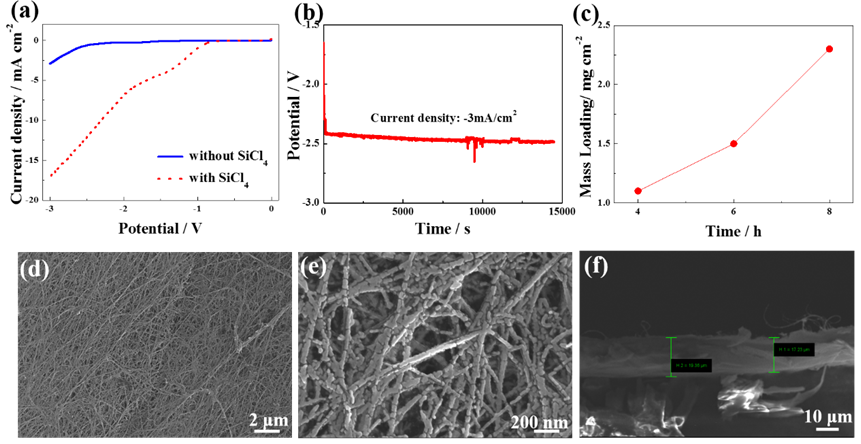


Fig. S1 (a) Linear sweep voltammograms of the three-electrode system with and without SiCl4 in the electrolyte. (b) Time-potential curve under constant current density of -3 mA cm-2. (c) The mass loading of the silicon deposit at different deposited time. SEM images taken from Si/CNTs-6 at (d) low magnification and (e) high magnification. (f) Cross-section SEM image of the Si/CNTs composite.

**
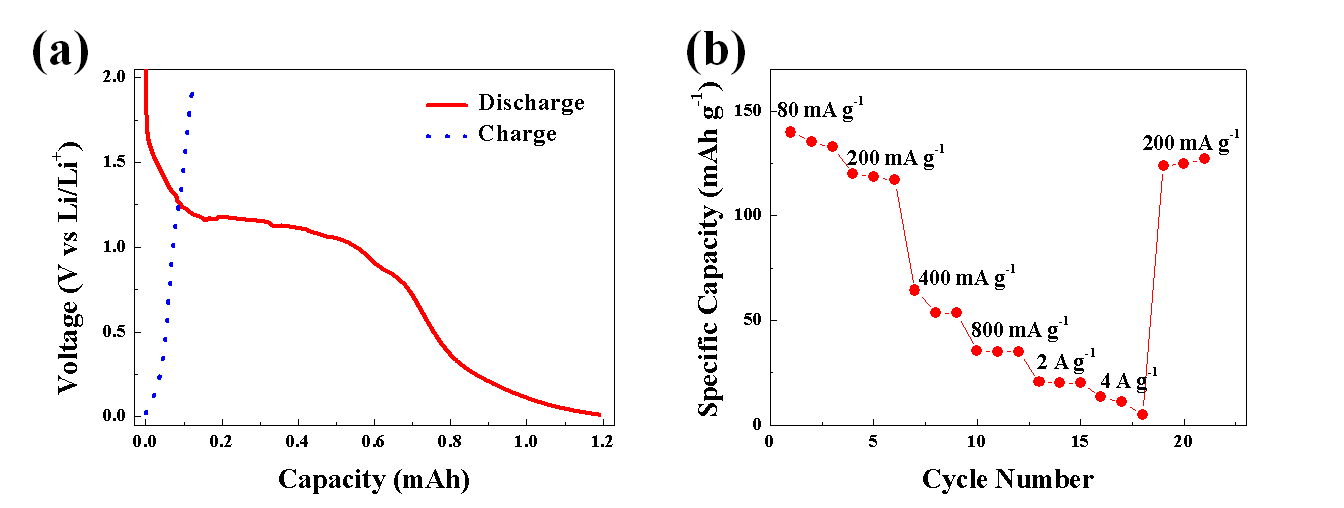
**

Fig. S2 (a) Voltage profile of the pristine CNTs electrode during the first cycles under the current density of 80 mA g-1. (b) The charge capacities of the pristine CNTs electrode at different rates.


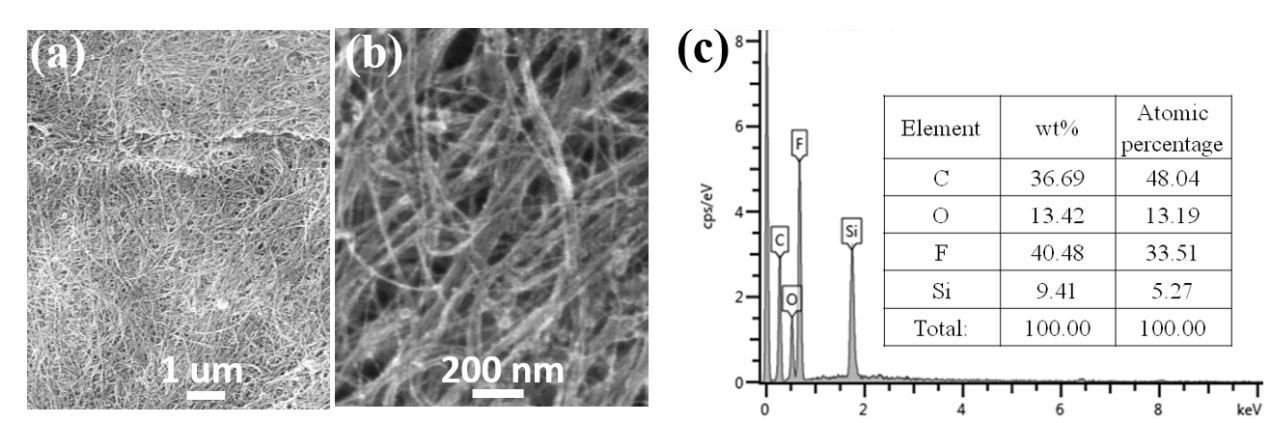


Fig. S3 (a) Low magnified, (b) high magnified plan-view SEM image taken from of the Si/CNTs-4 sample after 100 cycles tested. (c) EDX spectrum taken from this composite.

**Fig. S4** Comparison of specific capacities of the pristine and prelithiated Si/CNTs-4 at the various current densities.





Fig.S5 The discharging/charging curves of the full pouch cell with LiFePO_4_ cathode and Si/C anode at current density of 170 mA g^-1^ under flat and bent states.

**Table S1** Comparison of the volumetric capacities and other characteristic of some representative flexible Si-based lithium-ion battery anodes.

| Materials | structure | method | Mass loading  (mg/cm^2^) | Thicknes of active material  (μm) | Thickness of electrode  (μm) | Volumetric capacity mAh/cm^3^  (current density mA/g) | Specific capacity mAh/g^3^  (current density mA/g) | Strain/Stress  (%/Mpa) | Ref |
| --- | --- | --- | --- | --- | --- | --- | --- | --- | --- |
| Si/CNTs sheets | Core-shell  NW array | CVD | 0.3-0.4 | 0.1-0.2 | 100 | 54(400) | ~1250 | -- | [1] |
| VACNT-Si/CC | Si deposited on VACNT grown on CC | PECVD | 0.79 | <0.65 | ~0.41 | ~80(253) | <194(253) | -- | [2] |
| SiNP/CNT/CNC paper | Si NPs mixed with CNTs | Vacuum filtering | 0.79 | -- | 30 | 845(200) | ~800 | 1.3/27 | [3] |
| Si/Ni/PVDF | Si coating on flexible Ni/PVDF | Magnetic sputtering | 0.08 | 0.08 | 100 | 26(800) | ~320 | 17/2.5 | [4] |
| Si/C fiber paper | Si NPs in C fibers | Electrospray | 0.6-1.2 | -- | 14-30 | 850(400) | 1500(400) | -- | [5] |
| Si/Graphene Paper | sandwich-like | Etching and vacuum filtration | 0.5 | -- | 40 | ~188(100) | 1500(100) | -- | [6] |
| Si/PEDOT:PSS/CNT film | three-dimensionally interconnected hybrid hydrogel system | aqueous solution process | 1.14 | -- | 20 | 1100(420) | 2180(420) | -- | [7] |
| 4 layed Si/CNT | -- | CVD | 0.28 | -- | 20 | 625(840) | 2912(840) | -- | [8] |
| Si/CNT paper | Paper-like | pulsed laser deposition | -- | 3.5 | 93.5 | 107(54 μA cm^-2^) | -- | -- | [9] |
| CNT-Si film | Paper-like | CNTs are infiltrated in a Si  film | 0.2 | -- | 4 | 1000(360) | 2000(360) | -- | [10] |
| Si/CNTs-4 | Paper-like | Electrodeposition | 1.1 | -- | 20 | 932(200) or  823(400) | 1736(200) | 4.3/62 | This work |
| Si/CNTs-6 | Paper-like | Electrodeposition | 1.5 | -- | 20 | 1400(200) or  1201(400) | 1857(200) | -- | This work |

Note: NW: nanowire; CVD: chemical vapor deposition; VACNT:vertically aligned carbon nanotube; CC: carbon clith; PECVD: plasma enhanced chemical vapor deposition; NP: nanoparticle; CNC: cladophora nanocellulose; PVDF: Poly(vinylidene fluoride).

References

[1] K. Fu, O. Yildiz, H. Bhanushali, Y. Wang, K. Stano, L. Xue, X. Zhang, P.D. Bradford, Aligned carbon nanotube-silicon sheets: A novel nano-architecture for flexible lithium ion battery electrodes, Adv. Mater. 25 (2013) 5109-5114.

[2] X. Wang, L. Sun, R.A. Susantyoko, Q. Zhang, A hierarchical 3D carbon nanostructure for high areal capacity and flexible lithium ion batteries, Carbon 98 (2016) 504-509.

[3] Z. Wang, C. Xu, P. Tammela, J. Huo, M. Strømme, K. Edström, T. Gustafsson, L. Nyholm, Flexible freestanding Cladophora nanocellulose paper based Si anodes for lithium-ion batteries, J. Mater. Chem. A 3 (2015) 14109-14115.

[4] Q. Xiao, Q. Zhang, Y. Fan, X. Wang, R.A. Susantyoko, Soft silicon anodes for lithium ion batteries, Energy Environ. Sci. 7 (2014) 2261.

[5] Y. Xu, Y. Zhu, F. Han, C. Luo, C. Wang, 3D Si/C fiber paper electrodes fabricated using a combined electrospray/electrospinning technique for Li-ion batteries, Adv. Energy Mater. 5 (2015) 1400753.

[6] H. Jiang, X. Zhou, G. Liu, Y. Zhou, H. Ye, Y. Liu, K. Han, Free-standing Si/graphene paper using Si nanoparticles synthesized by acid-etching Al-Si alloy powder for high-stability Li-ion battery anodes, Electrochim. Acta 188 (2016) 777-784.

[7] Z. Chen, J.W.F. To, C. Wang, Z. Lu, N. Liu, A. Chortos, L. Pan, F. Wei, Y. Cui, Z. Bao, A three-dimensionally interconnected carbon nanotube-conducting polymer hydrogel network for high-performance flexible battery electrodes, Adv. Energy Mater. 4 (2014) 1400207.

[8] Q. Xiao, Y. Fan, X. Wang, R.A. Susantyoko, Q. Zhang, A multilayer Si/CNT coaxial nanofiber LIB anode with a high areal capacity, Energy Environ. Sci. 7 (2014) 655-661.

[9] E. Biserni, A. Scarpellini, A.L. Bassi, P. Bruno, Y. Zhou, M. Xie, High-performance flexible nanoporous Si-carbon nanotube paper anodes for micro-battery applications, Nanotechnology 27 (2016) 245401.

[10] Li-Feng Cui, Liangbing Hu, Jang Wook Choi, Y. Cui, Light-weight free-standing carbon nanotube-silicon films for anodes of lithium ion batteries, ACS Nano 4 (2010) 3671-3678.
